# A two-stage inversion technique for total auricular reconstruction: case report and literature review

**DOI:** 10.1186/s12893-018-0410-7

**Published:** 2018-09-18

**Authors:** Yanlu Lyu, Lingguo Ma, Chaoyang Ke, Wei Zhang, Ming Liu

**Affiliations:** Shenzhen People’s Hospital, 2nd clinical medical college of Jinan University, 1017, Dongmen north road, Luohu district, Shenzhen, 518020 Guangdong China

**Keywords:** Auricle amputation, Reconstruction, Two-stage inversion technique

## Abstract

**Background:**

The reconstruction of a total amputated auricle is aesthetically demanding for otorhinolaryngology surgeons. Although various reattachment methods have been introduced since 1898, only a few have achieved satisfactory aesthetic outcomes. This study aimed to present a successful case of auricular reconstruction using a two-stage inversion technique.

**Case presentation:**

The patient’s left ear was extensively lacerated in a violent event 3 h before admission. The first-stage surgery was performed within 6 h of ischemic time. The amputated segment was prepared and trimmed carefully, and the anterior aspect of the avulsed auricle was directly sutured. The posterior skin of the ear was separated from the cartilage to close the wound. Then, using an inversion maneuver, the cartilage was pushed into a postauricular underlying muscle bed. In the second-stage surgery 27 days after the first procedure, the auricle was released and the normal ear structure was restored using full-thickness skin grafting. During a follow-up of more than 9 years, the patient showed satisfactory postoperative results in terms of cosmetic and functional outcomes of the reattached auricle. The size of his left ear was about 90% of the size of his right ear.

**Conclusions:**

The reconstruction of a total amputated auricle is challenging. The key to surgery lies in the sufficient preservation of the meticulous shape of the cartilage. The microsurgical anastomosis is a good choice only in selected cases. It is believed that the two-stage inversion technique can be a simple alternative to reconstruct the auricle in most situations when it is lacerated and contaminated.

## Background

Traumatic total auricular amputation is a rare injury, and its reconstruction remains a surgical challenge due to the complex structure of the cartilage and limited blood supply. The loss of normal ear structure is a significant aesthetic and functional deformity, and the effect of treatments may influence the facial structure and life of patients forever. Therefore, surgeons should be meticulous with the selection of reconstruction methods. A variety of reconstruction techniques have been reported since the nineteenth century, including direct restitching, microvascular anastomosis, pocket methods, and secondary reconstructions. Each of these methods has limitations, such as procedure complexity and high failure rates. This study aimed to present a successful case of reconstruction using a two-stage inversion technique for a total amputated ear due to sharp instrument injury. This study was approved by the Human Ethics Committee of Shenzhen People’s Hospital. The patient had signed the informed consent.

## Case presentation

A 20-year-old man with no medical history was referred to the Ear, Nose, and Throat (ENT) emergency room at Shenzhen People’s Hospital on June 17, 2008. He had an extended laceration of his left ear by a shattered beer bottle during a violent fight 3 h ago.

**Fig. 1:**
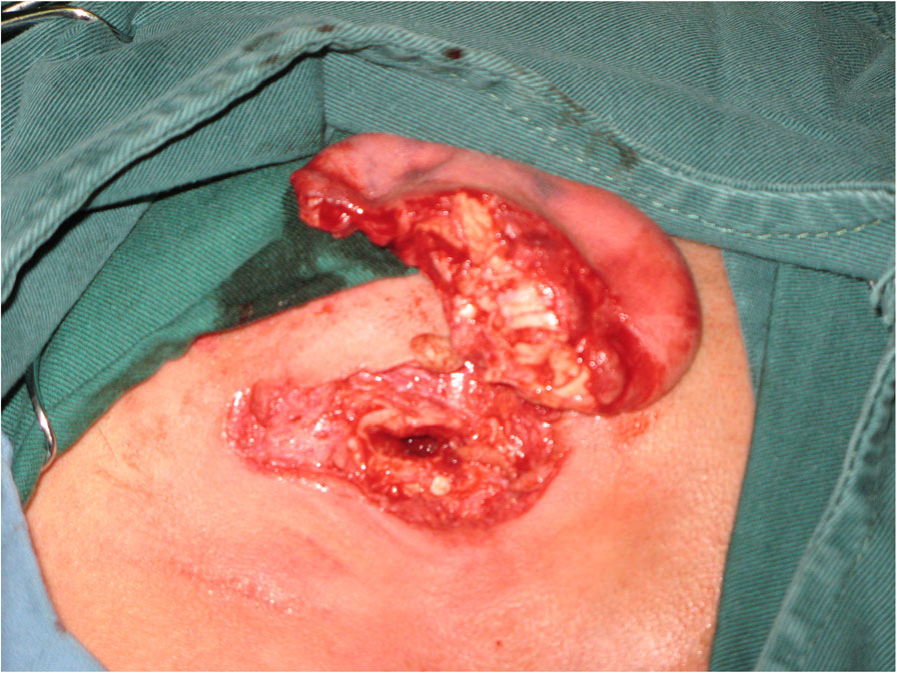
The left auricle was amputated completely, the external acoustic meatus was exposed. The skin was lacerated and the incisal margin of the cartilage was irregular

On examination, the external auditory canal of his left ear was amputated transversely, the tragus was lost, the cartilage was exposed, and the auricle was avulsed with only 5-mm skin attachment anteriorly at the crus of helix (Fig. 1). Other physical examinations were normal.

After informed consent was obtained, the patient was admitted and taken to the operation room within 3 h for the first-stage surgery under general anesthesia.

**Fig. 2:**
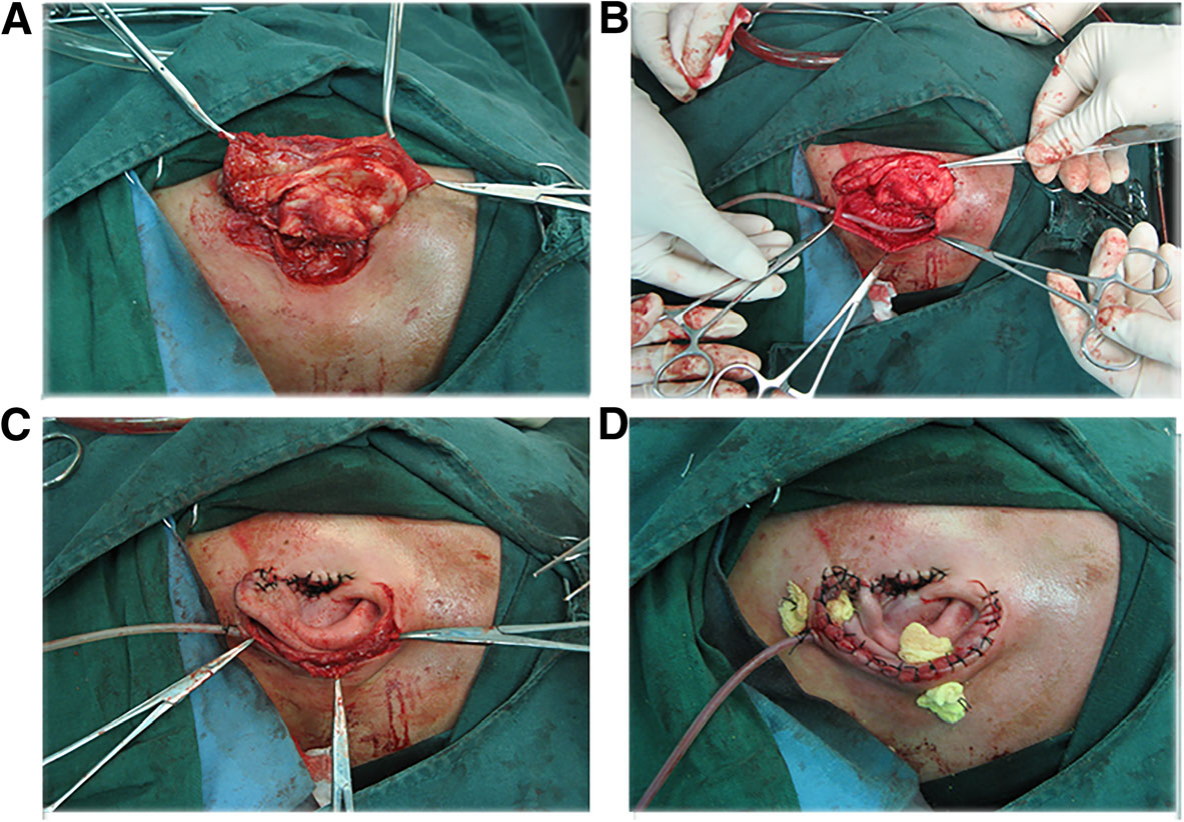
**a** The posterior skin on the auricle was separated from the cartilage. **b** The cut was prolonged, and the postauricular mastoid skin was elevated. **c** The margin and the free edge of the ear were sutured to close the wound. The cartilage and the inner side of the posterior skin of the auricle were pushed into the postauricular underlying muscle bed using an inversion maneuver. **d** A suction drain was placed, and the ear was packed with iodoform gauze and pressure bandage to strengthen the effect of inversion

First, the amputated ear segment was cleaned with saline, oxydol, and a diluted povidone-iodine solution. The irregular lacerated skin and cartilage were trimmed, and the anterior skin of the amputated segment and the external acoustic pore were sutured appositionally with multiple, interrupted No. 5–0 nylon sutures. Second, the skin on the posterior aspect of the amputated segment was separated from the cartilage with perichondrium preserved (Fig. 2a). Next, the wound was extended at the posterior sulcus of the auricle longitudinally by 1 cm upward and downward, and the postauricular mastoid skin was elevated about 1 cm to fit the size of the cartilage (Fig. 2b). Direct suturing was done between the margin and the free edge of the ear, and the wound was closed completely. Then, using an inversion maneuver, the cartilage and the inner side of the posterior skin of the auricle were pushed into the postauricular underlying muscle bed to provide nourishment and blood supply for the cartilage as extensively as possible (Fig. 2c). A suction drain was placed, and the ear was packed with iodoform gauze and pressure bandage to strengthen the effect of inversion (Fig. 2d).

Antibiotics consisting of intravenous cefuroxime 1.5 g every 12 h (GlaxoSmithKline Manufacturing S.p.A. Italy, with 1st dose given at the start of surgery), and intravenous metronidazole 0.5 g bid (Jiangsu Hengrui Medicine Co. Ltd., China) were both given for 1 week. Intravenous dextran 40 (Xian Wanlong Pharmaceutical Co. Ltd., China) infusions were given for 7 days. The patient was also given tetanus vaccine.

The nylon sutures were removed on postoperative day 7, and no external auditory canal stenosis or tissue necrosis was observed.

**Fig. 3:**
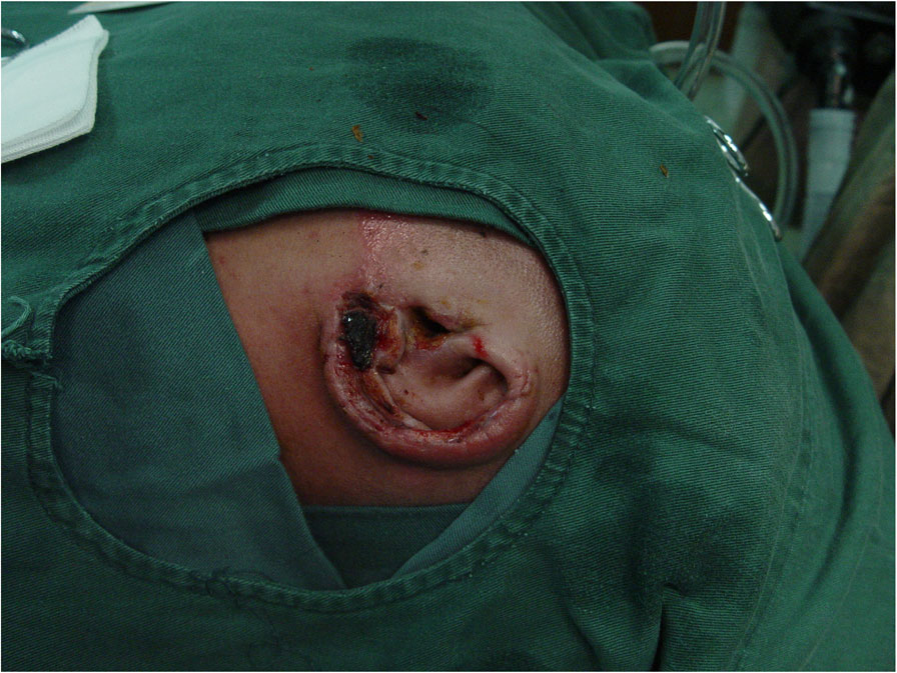
The left auricle healed well without skin ischemia or cicatricial stricture. Partial necrosis of the auricular lobule was found

The patient underwent a second-stage procedure 27 days after the initial surgery. On examination, the left auricle healed well without inflammation or skin ischemia; however, the auricular lobule had partial necrosis (Fig. 3).

**Fig. 4:**
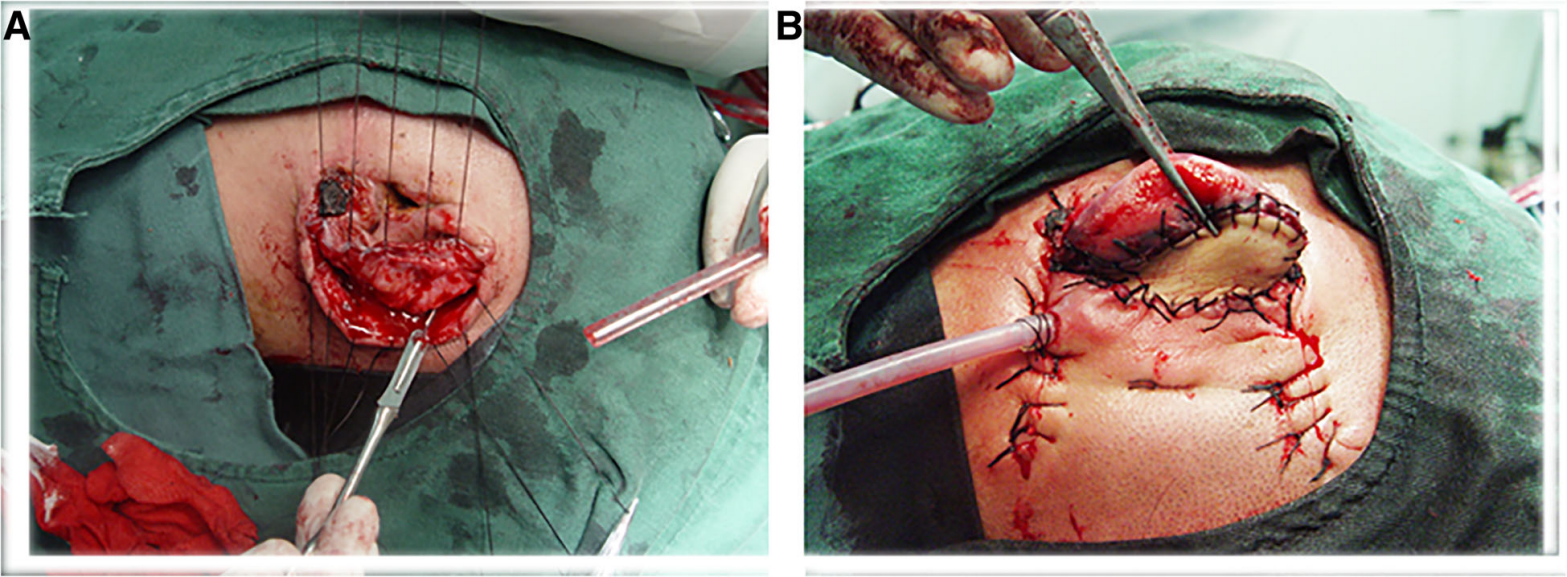
**a** The auricle was released, and the normal structure was restored. **b** The defect was reconstructed with a full-thickness skin grafting of the abdominal wall. A suction drain was placed

The auricle was released from the postauricular area, and the normal auricle structure was restored (Fig. 4a). Then, a skin defect (4 × 2 cm^2^) of the posterior aspect of the ear was noted, and a full-thickness skin grafting of the abdominal wall was applied to reconstruct it. A suction drain was placed, and the ear was covered with pressure bandage (Fig. 4b). The patient was given dextran 40 (Xian Wanlong Pharmaceutical Co. Ltd.) and vasodilators for 7 days.

**Fig. 5:**
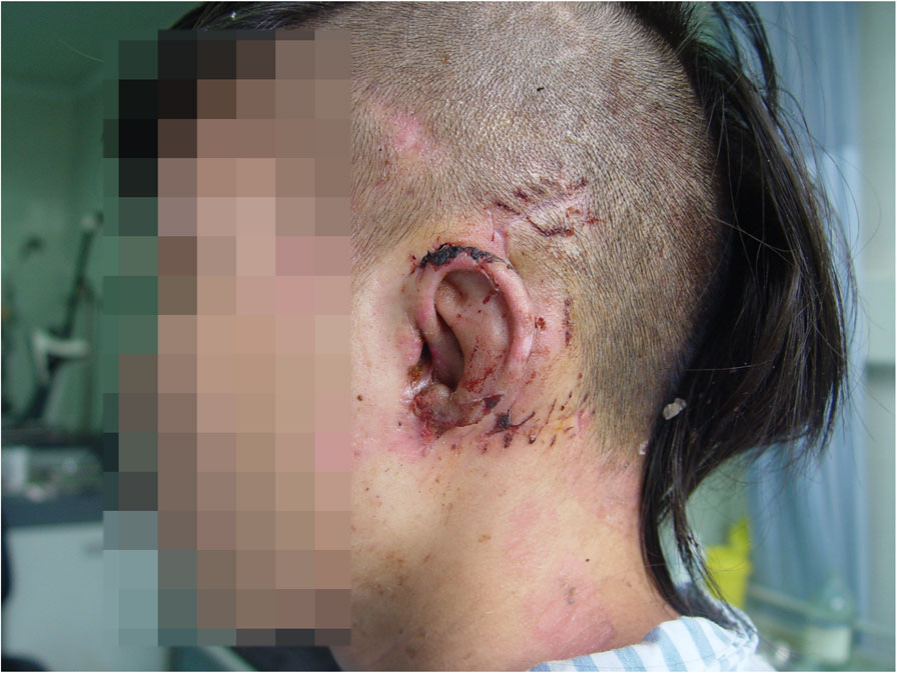
Good cosmetic appearance with minimal volume loss of the earlobe 15 days after the second-stage surgery

**Fig. 6:**
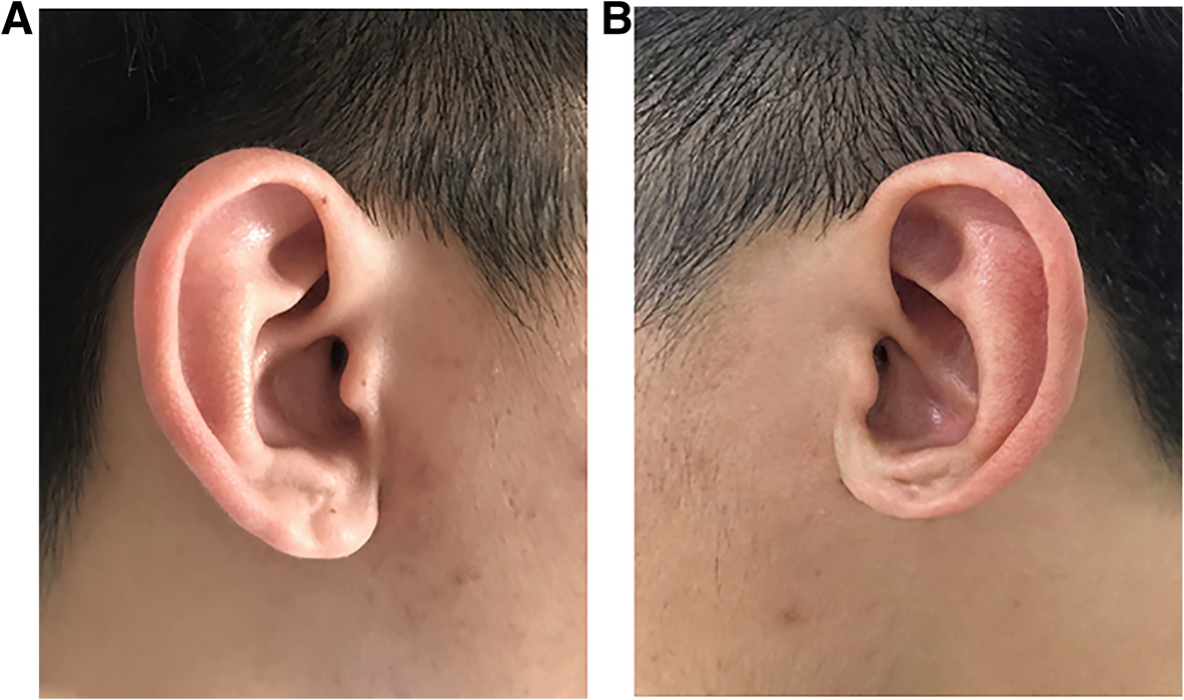
**a** The picture of the right auricle. **b** The left auricle continues to have good cosmetic and functional outcomes 10 years after surgery, with the size about 90% of the size of his right ear

The patient had a good recovery with minimal volume loss of the earlobe 15 days after the second-stage surgery (Fig. 5). The location and contour were normal. The color and temperature of the left ear were similar to those of the right one. The patient was followed up for nearly a decade, and continued to have good cosmetic and functional outcomes, with the size about 90% of the size of his right ear. The sensation and algesia were a little less sensitive (Fig. 6a, b).

## Discussion and conclusion

Successful reattachment of an avulsed auricle is a demanding task primarily due to the morphology and cartilage of the ear. Over the past years, many surgical approaches were used to reconstruct amputated auricles and maintain a satisfying aesthetic result.

The oldest report of ear reconstruction was by Brown in 1898 with direct reattachment of a horse-bite ear as a composite graft [1]. This technique was the simplest, but few successful cases of total amputations were reported. It was achieved when the amputated part was smaller than 15 mm in diameter [2]. Mladick introduced the “pocket principle” in 1971 [3] to improve the direct reattachment method; several variations of this technique have been described ever since. Their common characteristic is that the skin of the amputated part is removed by dermabrasion and then the avulsed cartilage is buried in a subcutaneous pocket, in either the postauricular, [4, 5] abdominal, [6, 7] supraclavicular, [8] cervical, [9] or forearm region [10, 11]. A second procedure to remove and re-epithelialize the buried pinna is required. Success has been reported to varying degrees using these methods. However, with total avulsed auricles, these techniques distort and flatten the contours of the cartilage, leading to an unsatisfying cosmetic outcome. In addition, it causes massive damages to the surrounding tissue.

Microsurgical ear replantation was first reported by Buncke in 1966 in an experimental model, [12] but the first clinical success was described in 1980 by Pennington [13]. The procedure involves one or more of the following techniques: vein grafts, primary vessel repair, arteriovenous shunts, or repair via pedicled superficial vessels [14]. In ideal situations, the microsurgical approach is considered to be the most promising procedure to save completely avulsed auricles. The success rate has been reported to be as high as 90% [15]. However, most cases of ear amputation involve avulsion, causing injury to the small vessels supplying the ear. As a result, finding appropriate vessels for anastomosis may be challenging. The use of vein graft would further increase the complexity as well as the time of operation. On the contrary, eminent disadvantages have been reported including the almost unavoidable need for blood transfusions to combat anemia, leeching, and anticoagulation therapy to dissolve postoperative venous congestion [1, 16]. In conclusion, when dealing with a traumatically totally severed auricle, microvascular replantation should be considered only in selected cases.

The proposed technique in situ involves embedding the cartilage in the postauricular region and enlarging the contact area of the severed cartilage with the muscle bed using the inversion maneuver, thus enhancing the nourishment and preserving its shape and function. The skin is removed but the perichondrium is preserved to reduce cartilage warping and flattening. The incision to elevate the mastoid skin is about 4 cm long, which minimizes the damage to the surrounding tissue and allows for secondary reconstruction in the case of failure.

The proposed method requires a second-stage surgery to release the buried part and reconstruct the normal shape of the auricle. The optimal timing is suggested as 1 month after the previous procedure, when the cartilage is fully revascularized. The technique of the second-stage surgery is adaptable due to the actual morphology of the auricle. Besides, the cleaning and preservation of the amputated segment before surgery and meticulous postoperative wound care are essential for survival. The use of antibiotics, dextran 40, and vasodilators are recommended.

The choice of surgical management of a total amputated auricle depends upon various factors, such as the size and location of the avulsed part, the condition of the segment and surrounding tissue, the ischemic time, the etiology of the injury, and the patient’s expectations. The proposed two-stage inversion technique showed a satisfactory long-term result while avoiding the complexity of the microsurgical method. Therefore, it might serve as a good choice in repairing a total amputated ear.

## Abbreviation

ENT: Ear, Nose, and Throat
